# Lats2 Modulates Adipocyte Proliferation and Differentiation via Hippo Signaling

**DOI:** 10.1371/journal.pone.0072042

**Published:** 2013-08-16

**Authors:** Yang An, Qianqian Kang, Yaofeng Zhao, Xiaoxiang Hu, Ning Li

**Affiliations:** State Key Laboratory for Agrobiotechnology, College of Biological Sciences, China Agricultural University, Beijing, China; Institute of Molecular and Cell Biology, Biopolis, United States of America

## Abstract

First identified in *Drosophila* and highly conserved in mammals, the Hippo pathway controls organ size. Lats2 is one of the core kinases of the Hippo pathway and plays major roles in cell proliferation by interacting with the downstream transcriptional cofactors YAP and TAZ. Although the function of the Hippo pathway and Lats2 is relatively well understood in several tissues and organs, less is known about the function of Lats2 and Hippo signaling in adipose development. Here, we show that Lats2 is an important modulator of adipocyte proliferation and differentiation via Hippo signaling. Upon activation, Lats2 phosphorylates YAP and TAZ, leading to their retention in the cytoplasm, preventing them from activating the transcription factor TEAD in the nucleus. Because TAZ remains in the cytoplasm, PPARγ regains its transcriptional activity. Furthermore, cytoplasmic TAZ acts as an inhibitor of Wnt signaling by suppressing DVL2, thereby preventing β-catenin from entering the nucleus to stimulate TCF/LEF transcriptional activity. The above effects contribute to the phenotype of repressed proliferation and accelerated differentiation in adipocytes. Thus, Lats2 regulates the balance between proliferation and differentiation during adipose development. Interestingly, our study provides evidence that Lats2 not only negatively modulates cell proliferation but also positively regulates cell differentiation.

## Introduction

Hippo signaling has emerged as an essential modulator of tissue and organ development. In mammals, the core of this pathway is a kinase cascade from the upstream kinase Mst1/Mst2 to the downstream effectors YAP/TAZ [1]. The upstream regulators, including NF2/Merlin, FRMD6/Ex1 and FRMD1/Ex2, are associated with cell junctions and are activated by various extracellular stimuli, such as cell contact, cell polarity and tension [2]. Upon activation, the extracellular signals are transduced to the kinases Mst1/Mst2, which are associated with Sav1/WW45. Then, Mst1/Mst2 phosphorylate and activate Lats1/2, two kinases that are regulated by MOB1A/1B. Following Lats1/2 activation, the transcriptional coactivators YAP and TAZ are phosphorylated and inactivated by Lats1/2, leading to their accumulation in the cytoplasm [3]–[5]. Under proliferating conditions, YAP and TAZ are unphosphorylated and associate with TEAD/TEF family transcription factors in the nucleus; these complexes can activate the expression of TEAD/TEF target genes, which promote cell proliferation and inhibit apoptosis. However, upon activation of Lats1/2, the expression of the target genes related to cell survival is inhibited due to the retention of YAP and TAZ in the cytoplasm [6]–[8]. Therefore, cell proliferation is repressed, and apoptosis is stimulated by Hippo signaling.

As a key component of the Hippo pathway, Lats2 plays major roles in cell proliferation and apoptosis and is an important regulator of tissue and organ development. For instance, Lats2 regulates the size of the heart and controls cardiac hypertrophy [9]. Because Lats2 is essential for tissue and organ size control, its down-regulation can cause tumorigenesis [10], [11]. Lats2 acts at the G1/S checkpoint to modulate cell cycle progression by inhibiting the G1/S transition [12]. Moreover, Lats2 plays an important role in mitosis by controlling the stabilization of mitotic regulators [13] and maintaining mitotic fidelity and genomic stability [14].

Although the regulation of the Hippo pathway is relatively well understood in many tissues and organs [15]–[18], less is known about the function of Lats2 and Hippo signaling in adipogenesis and adipose development. Adipose tissue is essential for the balance between energy intake and expenditure *in vivo*, and it acts as the major energy storage organ [19]. The excessive storage of energy as triglycerides can cause adipose tissue hypertrophy, leading to obesity, insulin resistance, type 2 diabetes and hyperlipidemia [20], [21]. Adipose tissue is derived from mesoderm and is composed mainly of adipocytes [22]. Adipose development involves preadipocyte proliferation and their differentiation into mature adipocytes. This differentiation occurs as the result of the synergistic effects of key transcriptional factors, especially peroxisome proliferator-activated receptor γ (PPARγ) and CCAAT/enhancer binding protein α (C/EBPα) [23]. Due to the importance of normal adipose development, it is necessary to determine the details of the mechanisms that regulate adipose development. Many genes and pathways are involved in modulating adipocyte proliferation and differentiation, such as Wnt [24], Hedgehog [25], MAPK [26], BMP and Smad signaling [27]. Although the mechanisms that modulate adipose development have been studied extensively, only a few of these studies have included Hippo signaling [28] or Lats2.

Here, we show that Lats2 is an important modulator of adipocyte proliferation and differentiation via the Hippo pathway that regulates the balance between proliferation and differentiation during adipose development.

## Materials and Methods

### Reagents

Dulbecco’s modified Eagle’s medium (DMEM), fetal bovine serum, bovine serum were obtained from Gibco (Gaithersburg, MD, USA). DAPI and PI were purchased from Beyotime (Nanjing, China).

### Cell Culture and Differentiation

The 3T3L1 cells (American Type Culture Collection, Rockville, MD) were maintained in DMEM supplemented with 10% bovine serum in 5% CO2 at 37°C [29]. Cells were passaged every third day. To induce differentiation, cells were exposed to an adipocyte differentiation cocktail (0.5 mM 3-isobutyl-1-methylxanthine, 5 µg/mL insulin, and 1 µM dexamethasone) after reaching confluence (day 0). At day 2, the differentiation medium was replaced with DMEM containing 10% fetal bovine serum and 10 µg/mL insulin. This medium was replaced with DMEM containing 10% fetal bovine serum every other day [30].

### DNA Constructs and Transfection

The mouse Lats2 CDS was obtained by PCR amplification using gene-specific primers and cloned into the pPB-CAG-EBNXN vector (kindly provided by Wellcome Trust Sanger Institute). Thus, the expression of the Lats2 coding sequence was driven by a CAG promoter, followed by a puromycin resistance gene. Cells were co-nucleofected with the pPB-CAG-EBNXN-Lats2 and pCAG-Base vectors using program T-030 on the Nucleofector (Amaxa) according to the manufacturer’s instructions. After 48 h, cells were selected with puromycin. Two weeks later, positive clones with stably integrated Lats2 were obtained.

### Quantitative RT-PCR

Total RNA was extracted from cells using the RNeasy Mini Kit (Qiagen, Valencia, CA, USA), and DNA was removed using the RNase-free DNase Kit (Qiagen) according to the manufacturer’s protocol. cDNA was synthesized using M-MLV reverse transcriptase (Promega, Madison, WI, USA). Quantitative RT-PCR was performed on a LightCycler® 480 Real-Time PCR System using SYBR Green I mix (Roche Molecular Biochemicals, Mannheim, Germany) according to the manufacturer’s instructions. The expression results were normalized to GAPDH.

### Western Blotting

Western blotting was performed on total proteins extracted from cells. The antibodies used for Western blotting were anti-Lats2 (Abcam, Cambridge, UK), anti-YAP (Cell Signaling Technology), anti-p-YAP (Cell Signaling Technology), anti-TAZ (BD Biosciences, San Jose, CA, USA), anti-p-TAZ (Santa Cruz Biotechnology, Santa Cruz, CA, USA), anti-CTGF (Santa Cruz Biotechnology), anti-survivin (Cell Signaling Technology), anti-cyclin E1 (Cell Signaling Technology), anti-p-DVL2 (Epitomics, Burlingame, CA, USA), and anti-α-tubulin (Beyotime).

### Immunofluorescence

For immunofluorescence analysis, cells were fixed with 4% paraformaldehyde in PBS for 30 min. The fixed cells were washed 3 times with PBS for 5 min. After washing, cells were treated with 0.3% Triton X-100 in PBS for 15 min. Permeated cells were washed as described above. Then, cells were incubated with PBS containing 2% BSA (blocking buffer) for 30 min and incubated successively with the appropriate primary and labeled secondary antibodies in blocking buffer for 1 h. Then, cells were stained with DAPI, and fluorescence was observed under a confocal microscope.

### MTS Cell Proliferation Assay

Cell growth rates were assessed using a CellTiter Aqueous MTS System (Promega) according to the manufacturer’s instructions. MTS is a tetrazolium compound that is bioreduced by cells into a colored formazan product; the amount of formazan produced by cells is proportional to the number of living (growing) cells. In brief, cells were plated at 10^3^ cells/well in 96-well culture plates. The time at which cells attached was designated as 0 h. Cell proliferation was assessed by MTS assay at 0 h, 24 h, 48 h and 72 h as described [31]. The absorbance was recorded at 490 nm.

### BrdU Cell Proliferation Assay

Cellular DNA synthesis rates were analyzed using the BrdU Cell Proliferation Assay Kit (Cell Signaling Technology) according to the manufacturer’s protocol. BrdU is a pyrimidine analog that is incorporated in place of thymidine into the newly synthesized DNA of proliferating cells. The magnitude of incorporated BrdU acts as a direct indicator of the number of S phase cells and degree of cell proliferation. Briefly, cells were plated in 96-well culture plates at 500 cells/well. The time at which cells attached was designated as 0 h. Cells were assessed by BrdU incorporation at 0 h, 24 h, 48 h, and 72 h. At the designated times, BrdU (10 µM) was added to each well. After 2 h, the medium was replaced with fixing/denaturing solution, and then cells were incubated with anti-BrdU antibody for 1 h. The incorporated BrdU was measured by the HRP-conjugated antibody to BrdU and TMB substrate. The absorbance was read at 450 nm.

### Cell-cycle analysis by Flow Cytometry

For cell-cycle analysis, cells were cultured in 10-cm dishes for 48 h. Cells were trypsinized and washed twice with PBS, then resuspended in 70% ethanol for 1 h at 4°C. Fixed and permeated cells were collected by centrifugation, washed twice with PBS, treated with RNase A, stained with PI, and measured by flow cytometry as described [31]. PI is a fluorescent intercalating agent that is capable of binding to DNA, and the G1, G2 and S phases can be distinguished by PI staining followed by flow cytometry.

### Luciferase Reporter Assay for Wnt/β-catenin Signaling

For the luciferase reporter assay, cells were transfected with the TOPflash or FOPflash plasmid and pRL-TK vector (*pRenilla* as internal control). The TOPflash plasmid contains TCF-binding sites and a luciferase CDS region, whereas the FOPflash plasmid contains mutant TCF-binding sites and thus serves as a negative control. After 24 h, cells were treated with or without Wnt3a (50 ng/ml) for 16 h as described [32]. Luciferase activity was assessed using the Dual-Glo Luciferase Assay System (Promega) according to the manufacturer’s protocol.

### Luciferase Reporter Assay for aP2 Promoter Activity

For aP2 promoter activity assay, aP2 promoter and PPARγ CDS were respectively cloned into pGL3-Basic vector (Promega) and pcDNA3.1 vector (Invitrogen Life Technologies, Groningen, the Netherlands), and then cells were co-transfected with pGL3-Basic-aP2-Promoter plasmids, pcDNA3.1-PPARγ plasmids or pcDNA3.1 empty vectors and pRL-TK vectors (*pRenilla* as internal control). After 24 h, cells were treated with or without Rosiglitazone (10 µM) for 24 h as described [33], and then the luciferase activity was measured using the Dual-Glo Luciferase Assay System (Promega).

## Results

### 1. Lats2 Enhances the Phosphorylation and Cytoplasmic Accumulation of YAP and TAZ in Preadipocytes

We used 3T3L1 cells to investigate the modulation of adipose development. This cell line is often used as a model for studies of adipose development because the behavior of 3T3L1 cells *in vitro* is similar to that of preadipocytes *in vivo* [19].

To identify the function of Lats2 in adipose development, we overexpressed Lats2 gene in 3T3L1 cells. At the protein level, the level of Lats2 and phosphorylated Lats2 (p-Lats2, active form) in Lats2-transfected cells was significantly higher than that in both control (Vector and Control) cells (Fig. S1A). Next, we assessed the cellular localization of Lats2 by immunofluorescence (IF). Confocal micrographs indicated that Lats2 localized predominantly to the cytoplasm with some nuclear localization in 3T3L1 cells (Fig. S1B). Together, these data suggest that the Lats2-overexpressing 3T3L1 cells have high levels of phosphorylated Lats2 and predominantly cytoplasmic Lats2 localization.

**Figure 1:**
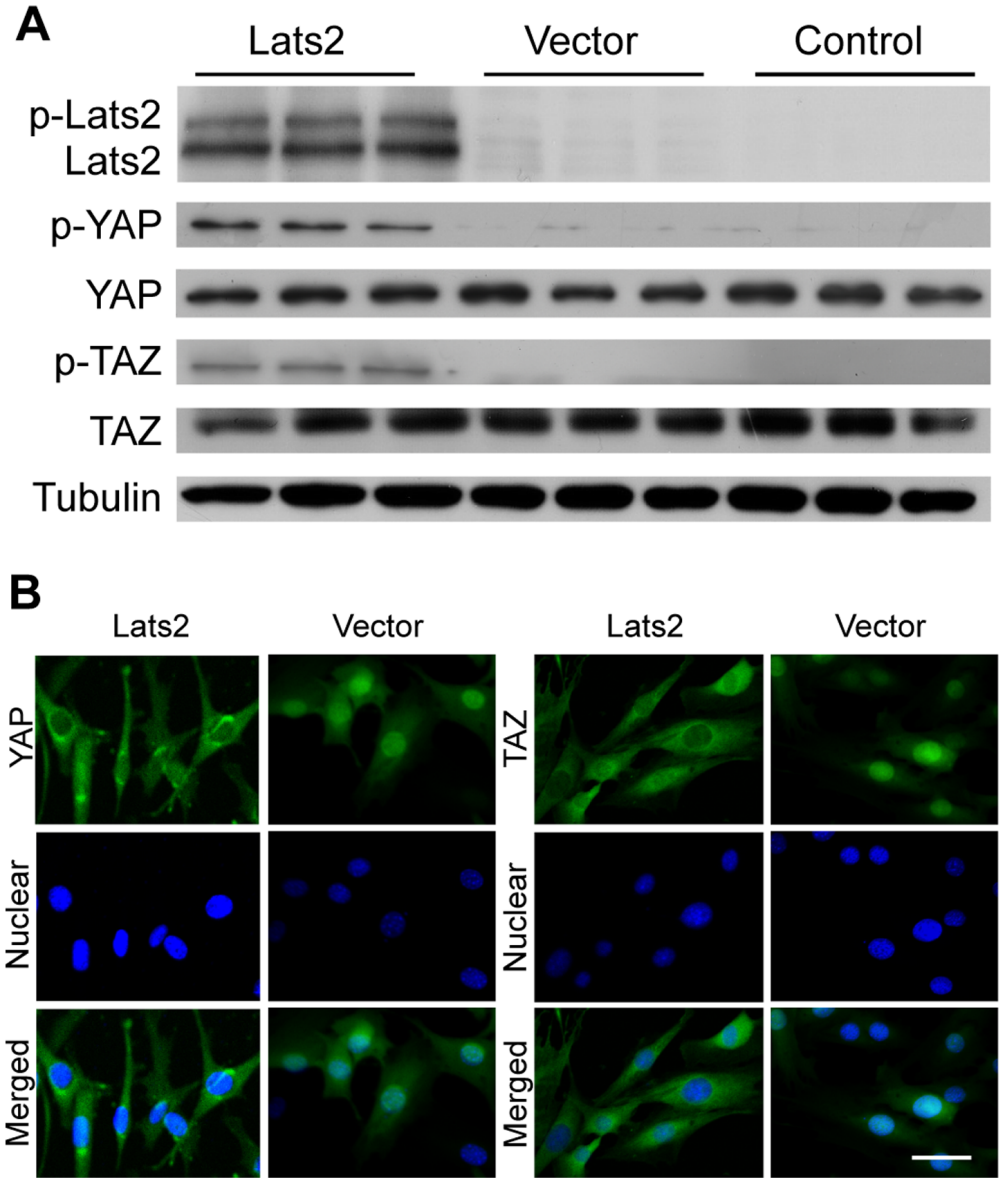
YAP and TAZ are phosphorylated by Lats2 and accumulate in the cytoplasm. (A) Lats2-mediated enhanced phosphorylation of YAP and TAZ. Whole-cell lysates were prepared from Lats2-transfected cells, immunoblotted with Lats2, p-YAP, YAP, p-TAZ, TAZ and Tubulin antibodies. (B) Lats2-mediated enhanced cytoplasmic accumulation of YAP and TAZ. Micrographs depict YAP and TAZ in 3T3L1 cells, as detected by anti-YAP and anti-TAZ antibodies (green). The nucleus was stained by DAPI (blue). The scale bar represents 20 µm.

YAP and TAZ are the downstream targets of Lats2 in the Hippo signaling pathway. Upon activation, Lats2 phosphorylates YAP and TAZ, leading to their retention in the cytoplasm [3]. Thus, we assessed the effects downstream of Lats2 in 3T3L1 cells. The levels of phosphorylated YAP and TAZ in Lats2-transfected cells were markedly higher than that in both control (Vector and Control) cells. No significant change in the total protein level of YAP or TAZ was observed (Fig. 1A). Confocal micrographs indicated that YAP and TAZ were accumulated in the cytoplasm in Lats2-transfected cells, yet they were localized in the nucleus in Vector cells (Fig. 1B). These results indicate that the downstream effectors of the Hippo pathway, YAP and TAZ, are phosphorylated by Lats2, leading to their retention in the cytoplasm of preadipocytes.

### 2. Proliferation of Preadipocytes is Repressed by Lats2

**Figure 2:**
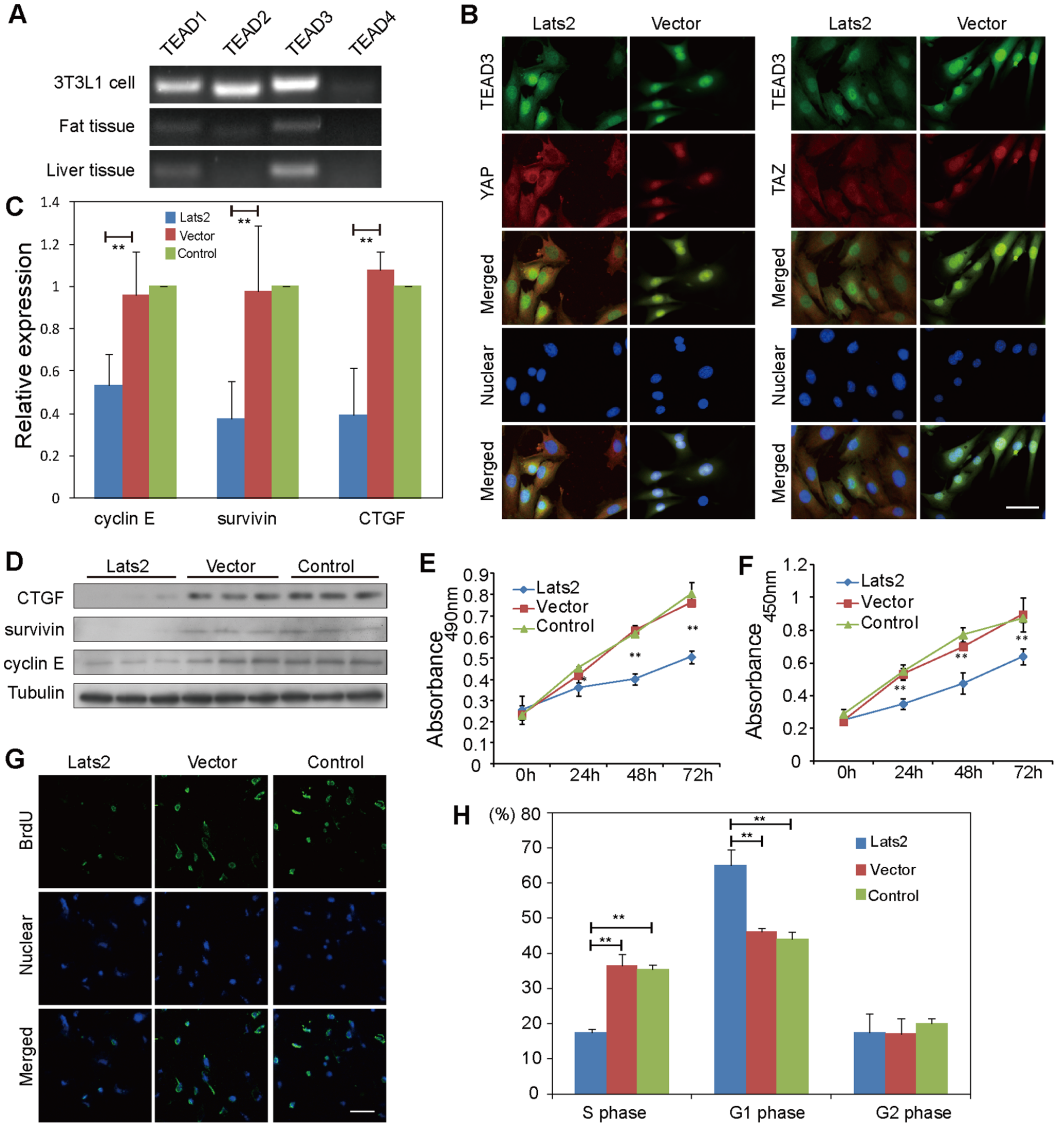
Lats2-mediated repressed proliferation of 3T3L1 preadipocytes. (A) TEAD3 is the main TEAD expressed in 3T3L1 cells, fat and liver tissue. RT-PCR assay was performed using TEAD1–4 specific primers. (B) TEAD3 localizes to the nucleus, but YAP and TAZ remain in the cytoplasm due to phosphorylation by Lats2. Micrographs depict TEAD3 in 3T3L1 cells as detected by anti-TEAD3 antibody (green). Anti-YAP and anti-TAZ antibodies appear red. The nucleus was stained by DAPI (blue). The scale bar represents 20 µm. (C) Lats2-mediated decrease of Hippo target gene expression at the mRNA level. Target gene transcript levels were measured by quantitative RT-PCR. The data shown are the means+S.D. of three independent experiments. (D) Lats2-mediated decrease of Hippo target gene expression at the protein level. (E) Preadipocytes growth is inhibited by Lats2. Cells were cultured in 96-well culture plates and treated with MTS at the designated times (every 24 h). After incubation, the absorbance was recorded at 490 nm. (F) Preadipocytes proliferation is delayed by Lats2. Cells were cultured in 96-well culture plates and treated with BrdU at the designated times (every 24 h). After incubation with BrdU antibody and substrate, the absorbance was read at 450 nm. (G) Lats2-mediated less DNA synthesis of preadipocytes. Micrographs show the BrdU incorporated in 3T3L1 cells as detected by anti-BrdU antibody (green). Cell nuclei were stained by DAPI (blue). The scale bar represents 20 µm. (H) Cell cycle progression of preadipocyte is delayed by Lats2. Cells were cultured in 10-cm dishes for 48 h and then stained by PI for flow cytometry. Statistics from three separate experiments showing the percentages of cells in G_1_, G_2_ and S phase, respectively. In (C), (E), (F) and (H), *P*-values were calculated using the Student’s t-test (*, *P*<0.05; **, *P*<0.01).

Given the above results, we next investigated the phenotypic effects caused by Lats2 in 3T3L1 cells. The TEAD/TEF transcriptional factors are the ultimate effectors of the Hippo pathway; the expression of Hippo target genes is regulated by the binding between TEAD and YAP/TAZ. Mammals express four TEAD proteins (TEAD1–TEAD4), and they are all widely involved in regulating development [34]. In our study, PCR showed that TEAD3 was the main form expressed in 3T3L1 cells, fat tissue and liver tissue (Fig. 2A), indicating that TEAD3 is possibly most responsible for initiating transcription in adipocytes. Therefore, we focused on TEAD3 to assess its interaction with YAP/TAZ. As shown in the confocal micrographs, TEAD3 and YAP/TAZ displayed predominantly nuclear co-localization in Vector cells, while YAP/TAZ displayed predominantly cytoplasmic localization in Lats2-transfected cells, indicating that these proteins might have been unable to form the transcriptional activation complex (Fig. 2B). Many target genes are modulated by Hippo signaling, including survivin (Baculoviral IAP repeat-containing 5, BIRC5) [35], connective tissue growth factor (CTGF) and cyclin E [7]. These genes are all involved in cell proliferation. As expected, the expression of cyclin E, survivin and CTGF all decreased in Lats2-transfected cells at both the mRNA and protein levels (Fig. 2C, D). The expression of survivin and CTGF was reduced to approximately 38% of their normal levels, while cyclin E decreased to 53% (Fig. 2C). These results indicate that the expression of Hippo target genes is suppressed due to YAP and TAZ retention in the cytoplasm caused by Lats2.

Because these Hippo target genes promote cell proliferation, we further investigated the cell cycle and growth rate in Lats2-transfected 3T3L1 cells through MTS and BrdU assays and cell-cycle analysis. For the MTS assay, cells were treated with MTS every 24 h. From 0 h to 72 h, Lats2-transfected cells exhibited delays in growth. At 72 h, both control cells (Vector and Control) had reached confluence, but the growth of Lats2-transfected cells was still inhibited (Fig. 2E). To further confirm the effect of Lats2 activation on cell growth and the cell cycle, we measured BrdU incorporation every 24 h. By 48 h, Lats2-transfected cells displayed significantly less DNA synthesis than both control cells, and they began to proliferate at approximately 72 h (Fig. 2F). To observe cell proliferation more directly, we performed IF analysis using the BrdU antibody. As shown, the magnitude of incorporated BrdU in Lats2-transfected cells was visibly lower than that in both control cells (Fig. 2G), indicating that cell proliferation and cell cycle progression were delayed by Lats2. We next analyzed the cell cycle by flow cytometry using propidium iodide (PI). As shown, the percentage of Lats2-transfected cells in G1 phase was much higher than the percentage for both control cells. However, the percentage of Lats2-transfected cells in S phase was significantly lower than those in both control groups (Fig. 2H). These analyses reveal that the G1/S transition is strongly inhibited in Lats2-transfected cells, leading to G1/S arrest.

Taken together, the above observations confirm that upon Lats2 activation, YAP and TAZ are inactivated and sequestered in the cytoplasm, where they cannot form a complex with TEAD, leading to the down-regulation of downstream target genes. Therefore, the cell cycle progression and proliferation of preadipocytes are significantly inhibited by Lats2 via Hippo signaling.

### 3. Lats2 is a Positive Modulator of Adipocyte Differentiation

Although the function of Lats2 in cell proliferation is well known, surprisingly less is known about whether Lats2 plays a major role in cell differentiation. After investigating the effect of Lats2 on preadipocyte proliferation, we sought to determine the function of Lats2 in adipocyte differentiation. Adipogenic differentiation is controlled by a transcriptional cascade that involves a temporally regulated series of gene-expression events in which PPARγ is a core factor [36]. After receiving the signal to differentiate, Krox20 activates C/EBPβ, which then induces the expression of the key transcription factor PPARγ [37]. Next, PPARγ stimulates its own expression and that of C/EBPα, and C/EBPα in turn facilitates the further expression of PPARγ. PPARγ is also induced by Krüppel-like factor 5 (KLF5), the expression of which is also stimulated by C/EBPβ [38]. Upon activation, PPARγ initiates a strong adipogenic conversion [36], [39]. PPARγ and C/EBPα synergistically stimulate the expression of adipocyte marker genes, such as adipocyte protein 2/fatty acid-binding protein 4 (aP2/FABP4) [40], lipoprotein lipase (LPL) [41], fatty acid synthase (FAS) [40] and adiponectin [42], [43]. All of these genes contribute to the mature adipocyte phenotype [19].

**Figure 3:**
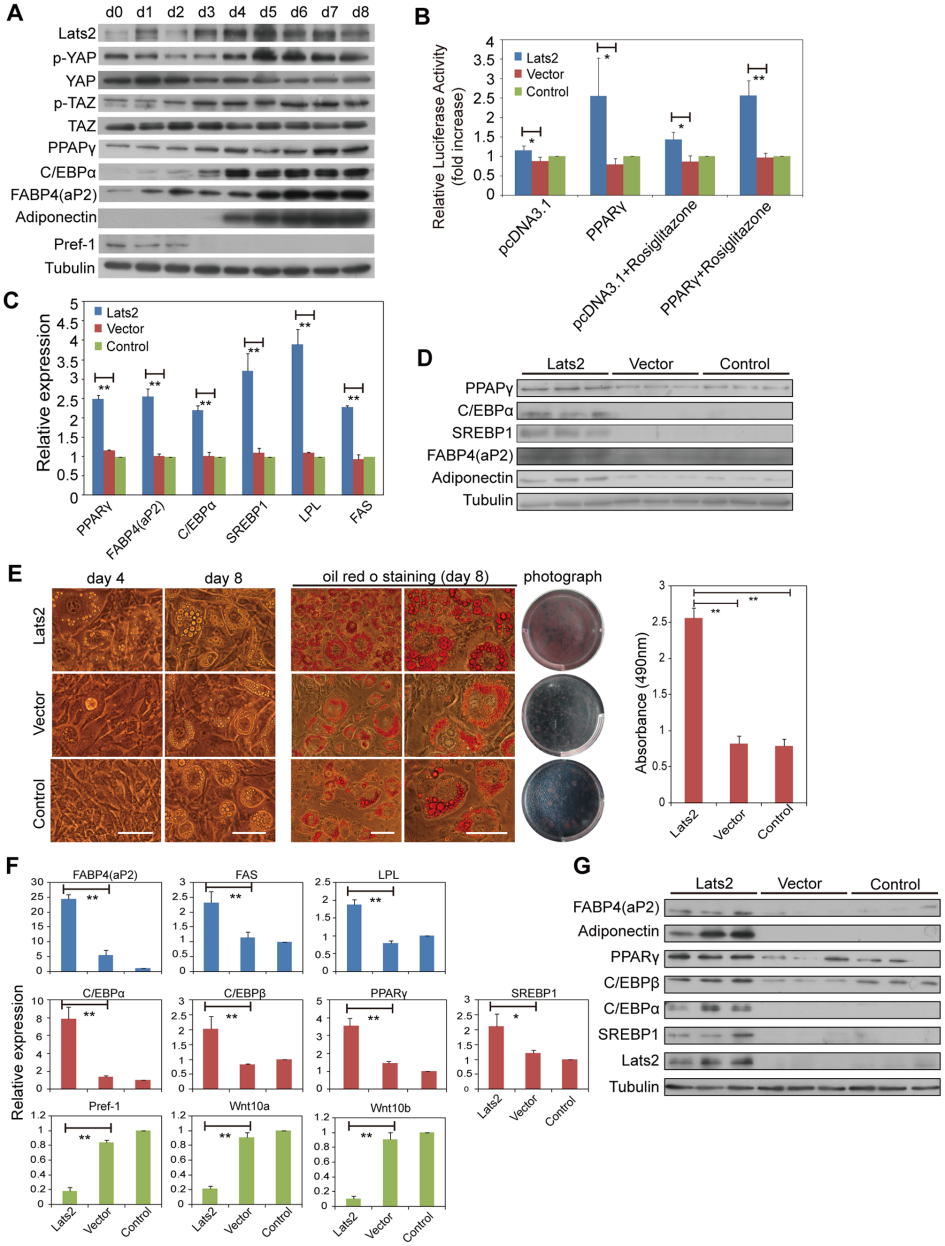
Adipocyte differentiation is promoted by Lats2. (A) Western blot analyses. Whole-cell lysates were prepared from differentiating 3T3L1 cells (day 0-day 8). (B) Lats2 enhances the transcriptional activity of PPARγ. Cells were co-transfected with pGL3-Basic-aP2-Promoter plasmids, pcDNA3.1-PPARγ plasmids or pcDNA3.1 empty vectors and pRL-TK vectors (*pRenilla* as internal control). After 24 h, cells were treated with or without Rosiglitazone (10 µM). pcDNA3.1 denotes pcDNA3.1 empty vector transfection, and PPARγ denotes pcDNA3.1-PPARγ transfection. (C) Lats2-mediated enhanced mRNA levels of SREBP1, PPARγ and its target genes. The data shown are the means+S.D. of three independent experiments. (D) Lats2-mediated enhanced protein levels of SREBP1, PPARγ and its target genes. (E) The differentiation of 3T3L1 cells is accelerated by Lats2. At day 4 and day 8 of adipocyte differentiation, 3T3L1 cells were observed under a microscope. At day 8, cells were stained with Oil Red O and photographed. The scale bar represents 20 µm. (F) and (G) Lats2-mediated enhanced expression of adipocyte marker genes in differentiating 3T3L1 cells. Total RNA and protein were isolated from the cells shown in (E) at day 4 for quantitative RT-PCR and Western blotting, respectively. In (B), (C), (E) and (F), *P*-values were calculated using the Student’s t-test (*, *P*<0.05; **, *P*<0.01).

To elucidate the relationship between Lats2 and adipocyte differentiation, we assessed the expression of Lats2 and other factors during 3T3L1 cells differentiation. The expression of Lats2 was up-regulated at the protein level (Fig. 3A), on the whole in parallel with that of the adipogenic transcriptional factors PPARγ and C/EBPα and the adipocyte marker genes aP2/FABP4 and adiponectin, while anti-adipogenic factors preadipocyte factor-1 (Pref-1) decreased, as expected (Fig. 3A). Pref-1 is an EGF repeat-containing protein that acts as a preadipocyte marker to inhibit cell differentiation and maintain the undifferentiated preadipocyte state [19]. At day 3, the protein level of Lats2 began to increase. As a result, the phosphorylation levels of YAP and TAZ increased from day 3 to day 8, indicating that the activities of YAP and TAZ were suppressed by Lats2 during 3T3L1 cells differentiation. In contrast, the protein levels of YAP and TAZ more or less decreased during differentiation (Fig. 3A). These results suggest that there must be some relationship between Lats2 and adipocyte differentiation, which Lats2 might regulate by interacting with YAP or TAZ to affect PPARγ, C/EBPα and Pref-1 or other factors.

TAZ functions as a transcriptional coactivator that regulates mesenchymal stem cell (MSC) differentiation [33]. TAZ, but not YAP, binds to and inactivates PPARγ, inhibiting adipocyte differentiation by transcriptionally suppressing PPARγ-driven gene expression [33]. Therefore, we next focused on TAZ to explore the mechanism by which Lats2 may modulate adipocyte differentiation. We speculated that Lats2 might relieve PPARγ from TAZ suppression by inactivating TAZ. To test this hypothesis, we assessed the transcriptional activity of PPARγ through luciferase activity assays. The pGL3-Basic-aP2-Promoter plasmid contains PPAR responsive element (PPRE) and a luciferase CDS region, so its luciferase activity serves as an indicator of PPARγ transcriptional activation capacity. As shown, the relative luciferase activity driven by aP2 promoter in Lats2-transfected cells is a little higher than that in both control (Vector and Control) cells, and it significantly increased by 2.5-fold in Lats2-transfected cells in the present of PPARγ (Fig. 3B). Upon Rosiglitazone stimulation, the relative luciferase activity increased by 1.44-fold in Lats2-transfected cells in the absent of PPARγ, and 2.6-fold in the present of PPARγ (Fig. 3B). These findings suggest that PPARγ retrieves its transcriptional activity through Lats2. Therefore, we assessed the expression of PPARγ-driven genes, including PPARγ itself, aP2/FABP4, C/EBPα, LPL, FAS and adiponectin, which are all involved in adipogenesis. We also assessed the expression of another adipogenic transcriptional factor sterol regulatory element-binding protein 1 (SREBP1) [36]. As expected, the expression of PPARγ, aP2/FABP4, C/EBPα, and SREBP1 significantly increased at both mRNA and protein levels in Lats2-transfected cells (Fig. 3C, D). The mRNA levels of LPL and FAS and the protein level of adiponectin increased as well (Fig. 3C, D). These results indicate that the expression of PPARγ-driven genes and adipogenic factor SREBP1 is promoted by Lats2.

Because these PPARγ target genes and SREBP1 contribute to adipogenesis, we next observed the phenotypic effects of Lats2 on adipocyte differentiation. We induced differentiation in 3T3L1 cells for 8 days. At day 4, adipocytes containing high levels of lipid droplets began to emerge in Lats2-transfected cells, but no evident morphological change occurred in both control cells (Vector and Control) (Fig. 3E). At day 8, as evidenced by staining with Oil Red O, a dye that specifically stains triglycerides, the Lats2-transfected cells had differentiated into mature adipocytes full of large triglyceride droplets, but both control cells displayed incomplete differentiation (Fig. 3E). The triglyceride content in Lats2-transfected cells was much higher than that in both control cells (Fig. 3E), indicating that the Lats2-transfected cells are significantly more mature than wild-type cells. At the mRNA level, the expression of the mature adipocyte markers aP2/FABP4, FAS and LPL in Lats2-transfected cells increased, so did the expression of the key transcriptional factors C/EBPα, C/EBPβ, PPARγ and SREBP1. In contrast, the expression of the adipogenesis inhibitors Pref-1, Wnt10a and Wnt10b decreased (Fig. 3F). Moreover, the protein levels of aP2/FABP4, adiponectin, PPARγ, C/EBPβ, C/EBPα and SREBP1 were all up-regulated by Lats2 (Fig. 3G). These findings reveal that Lats2 regulates adipogenesis by accelerating differentiation of preadipocytes into mature adipocytes.

Taken together, the above observations confirm that upon Lats2 activation, TAZ is inactivated and cannot enter the nucleus to bind and inhibit PPARγ. Therefore, PPARγ regains its transcriptional activity, leading to the expression of adipogenic genes. In short, Lats2 acts as an accelerator of adipocyte differentiation by inactivating TAZ.

### 4. Lats2 is a Negative Regulator of Wnt Signaling

The above results demonstrated the phenotypic effects of the Lats2 and Hippo pathway on adipocyte proliferation and differentiation and partially explained the mechanism by which Lats2 regulates adipose development. Next, we explored additional mechanisms of Lats2 action in preadipocytes. Many genes and pathways regulate adipocyte proliferation and differentiation, among which the Wnt pathway is essential. Wnt signaling maintains preadipocytes in the undifferentiated state by inhibiting the key adipogenic transcriptional factors PPARγ and C/EBPα [44]. Thus, Wnt signaling modulates adipose development by promoting preadipocyte proliferation and simultaneously inhibiting adipocyte differentiation [45]. Recent reports have connected the Wnt and Hippo pathways. Intriguingly, in the cytoplasm, TAZ suppresses the phosphorylation of DVL2, thereby inhibiting Wnt signaling [32]. Therefore, we next investigated whether Wnt signaling is inhibited in Lats2-transfected 3T3L1 cells, and if so, whether this mechanism also contributes to the phenotypes of repressed proliferation and enhanced differentiation in adipocytes.

The Wnt pathway plays many important roles in animal development. This pathway is composed of the extracellular Wnts and intracellular components such as the Frizzled (Fzd) receptors, low-density lipoprotein receptor-related protein (LRP) receptors, glycogen synthase kinase 3 (GSK3), disheveled (DVL), β-catenin, and T cell factor/lymphoid-enhancing factor (TCF/LEF). Upon the binding of a Wnt to its Fzd receptor and LRP co-receptor, DVL is phosphorylated, thereby releasing β-catenin from the GSK3-APC-axin destruction complex. Then β-catenin translocates to the nucleus to activate the TCF/LEF transcription factors, leading to the enhanced expression of Wnt target genes [24], [46]. The genes modulated by Wnt signaling include Pref-1 (also known as Delta-like1, DLK1) [47], LEF1 [48], cyclin D1 [49], c-Myc [50], survivin [51], and Axin [52], all of which are involved in cell proliferation. The adipogenic factors PPARγ and LPL are down-regulated by Wnt signaling [53].

**Figure 4:**
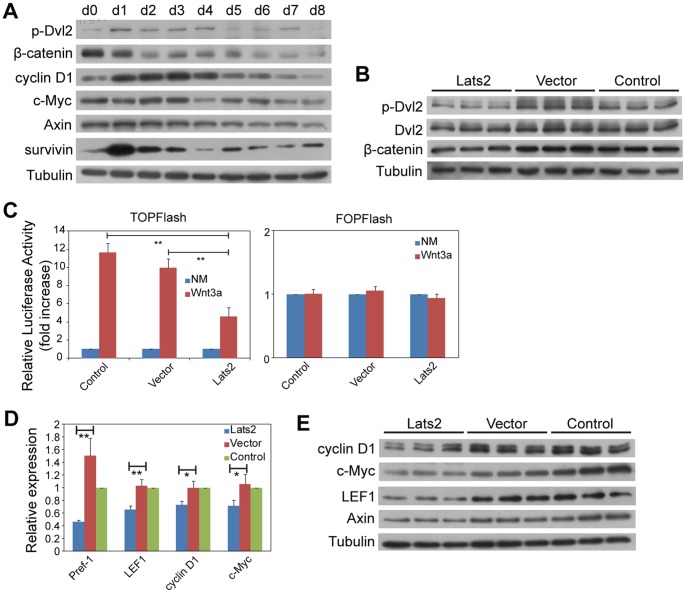
Lats2 inhibits Wnt signaling. (A) The protein levels of p-DVL2, β-catenin and Wnt signaling targets decrease during adipocyte differentiation. Total cell lysates were prepared from differentiating 3T3L1 cells. (B) Lats2-mediated decrease in β-catenin level. Western blot shows that the levels of DVL2 phosphorylation and β-catenin protein are both reduced by Lats2. (C) Lats2 suppresses the Wnt3a-induced activity of the TOPflash reporter. TOPflash and pRL-TK plasmids were co-transfected into Lats2-transfected cells and the two control (Vector and Control) cells, which were then treated with or without Wnt3a (50 ng/ml). The pRL-TK plasmid (*pRenilla*) was used as an internal control. The FOPflash assay was used as a negative control. NM, normal medium. (D) and (E) Lats2 inhibits Wnt target gene expression. Quantitative RT-PCR results from Lats2-transfected cells indicate that Pref-1, LEF1, cyclin D1, and c-Myc mRNA levels are reduced by Lats2. The data shown are the means+S.D. of three independent experiments. Western blot shows that the protein levels of cyclin D1, c-Myc, LEF1 and Axin are also reduced by Lats2. In (C) and (D), *P*-values were calculated using the Student’s t-test (*, *P*<0.05; **, *P*<0.01).

During 3T3L1 preadipocytes differentiation, at the protein level, the phosphorylation level of DVL2 was reduced, and total β-catenin decreased, so did the Wnt target genes cyclin D1, c-Myc, Axin and survivin, whereas the levels of Lats2, phosphorylated TAZ, PPARγ and C/EBPα increased (Fig. 4A and 3A). These results reveal that some Wnt pathway components and its target genes are inhibited during adipocyte differentiation; this inhibition might be mediated by TAZ, Lats2 or some other factors.

As described above, the phosphorylation of DVL2 controls the destruction of β-catenin, and TAZ can affect the phosphorylation of DVL2 [32]. As shown, the levels of DVL2 phosphorylation and thus β-catenin protein were reduced in Lats2-transfected cells (Fig. 4B). β-Catenin and TCF are the nuclear effectors of Wnt signaling [46], and the TCF family members such as TCF7L2 regulate adipose development [54]_ENREF_39. We next assessed whether Lats2 affects β-catenin’s ability to activate TCF-driven transcription through luciferase activity assays employing TOPflash/FOPflash plasmids. As shown, upon Wnt3a stimulation, the relative luciferase activity of TOPflash increased by approximately 11.6- and 10-fold, in Control and Vector, respectively, whereas the relative luciferase activity of TOPflash in Lats2-transfected cells only increased by approximately 4.6-fold (Fig. 4C Left). As expected, there was no significant difference in the relative luciferase activities of FOPflash upon Wnt3a stimulation (Fig. 4C Right). Therefore, Lats2 suppressed the TOPflash reporter activity induced by Wnt3a, indicating that the transcriptional activity of TCF was inhibited by Lats2. Thus, we next assessed the expression of Wnt target genes. At the mRNA level, the expression of the Wnt target genes Pref-1, LEF1, cyclin D1, c-Myc and survivin decreased in Lats2-transfected cells (Fig. 4D). Of note, survivin is a target gene not only of Hippo but also of Wnt signaling (Fig. 2C). Moreover, the adipogenic factors PPARγ and LPL are down-regulated by Wnt, but their expression was enhanced in Lats2-transfected cells (Fig. 3C). At the protein level, the expression of cyclin D1, c-Myc, LEF1 and Axin was reduced in Lats2-transfected cells (Fig. 4E). These findings indicate that Lats2 suppresses the expression of Wnt target genes, while the genes that are down-regulated by Wnt are up-regulated by Lats2.

Taken together, the above observations confirm that Lats2 inhibits Wnt signaling by phosphorylates TAZ. Our findings show that the inhibition of Wnt signaling and promotion of Hippo signaling are two mechanisms by which Lats2 contributes to the adipocyte phenotypes of repressed proliferation and enhanced differentiation.

## Discussion

Hippo pathway plays an important role in controlling tissue and organ size. This kinase cascade is regulated by cell adhesion, cell polarity and cell junction proteins [55]. Lats2 is one of the core kinases of the Hippo pathway and involved in modulating cell growth and survival by phosphorylating and inactivating transcriptional regulators YAP and TAZ [55]. Lats2 regulates diverse tissues and organs development, but Lats2 was first linked to adipose development in 2010 [56]. However, no direct evidence has shown that Lats2 can modulate adipose development.

The current investigation provided evidence that Lats2 is an important modulator of adipocyte proliferation and differentiation via Hippo signaling. Intriguingly, our research raises the possibility that Lats2 not only inhibits cell proliferation but also promotes cell differentiation. Hippo signaling involves a cytoplasmic kinase cascade [55], and the Lats2 protein is cytoplasmic during interphase in NIH3T3 cells but becomes localized to the mitotic apparatus during mitosis [12]. In our study, Lats2 was mostly localized to the cytoplasm around the nucleus, while a small amount localized to the nucleus, consistent with the function of Lats2 as a core component of Hippo signaling in the cytoplasm of 3T3L1 cells.

YAP and TAZ are downstream effectors of Hippo signaling that are regulated by Lats2, and they act as transcriptional co-activators of the TEAD/TEF family of transcription factors [1]. In our study, Lats2 enhances the phosphorylation and cytoplasmic accumulation of YAP and TAZ in preadipocytes, while TEAD3 proteins localized in the nucleus, indicating that YAP and TAZ are both inactivated by Lats2, leading to the suppressed transcriptional activity of TEAD3. Thus, the Hippo target genes (such as cyclin E, survivin and CTGF) expression was significantly suppressed by Lats2. Of note, cyclin D1 expression was also reduced by Lats2. Cyclin E and cyclin D1 are involved in regulating cell-cycle by promoting G1/S transition [57], [58]. Our data indicate that Lats2 inhibits cell cycle progression of preadipocytes mainly by blocking the G1/S transition. In summary, the proliferation and cell cycle progression of preadipocytes are suppressed by Lats2-mediated decreases of several regulators of cell growth.

Although the function of Lats2 in cell proliferation has been established, surprisingly less is known about whether Lats2 plays a major role in cell differentiation. Intriguingly, our investigation provided evidence that Lats2 is a positive modulator of adipocyte differentiation. It has been reported that TAZ, but not YAP, binds to PPARγ and directly inhibits the transcriptional activity of PPARγ, repressing adipocyte differentiation [33]. Recently, it has been reported that TAZ is downregulated by dexamethasone through glucocorticoid receptor (GR) during the differentiation of 3T3L1 preadipocytes [28], however, this paper did not mention the phosphorylation level of TAZ. Here, we show that the phosphorylation level of TAZ increased during adipocyte differentiation due to the enhanced protein level of Lats2. In our study, Lats2-mediated phosphorylation of TAZ leads to its retention in the cytoplasm such that it cannot enter the nucleus to bind PPARγ; thus, PPARγ regains its ability to activate pro-adipogenic genes. Our study indicate that Lats2 acts as an accelerator of adipocyte differentiation by inactivating TAZ, thus indirectly promoting PPARγ activity.

Together, our findings raise the possibility that Lats2 not only suppresses preadipocyte proliferation but also promotes preadipocyte differentiation. Nevertheless, we considered the possibility that Lats2 might act through more than one mechanism to regulate adipose development. Wnt signaling modulates adipose development by promoting preadipocyte proliferation and concomitantly inhibiting adipocyte differentiation [45]. Recently, reports on the crosstalk between Wnt and Hippo signaling have revealed that Hippo inhibits Wnt signaling by suppressing DVL2 through TAZ, leading to the destruction of β-catenin. In our study, we observed that the phosphorylation level of DVL2 and protein level of β-catenin both decreased by Lats2, and these might cause Lats2-mediated the repression of Wnt3a-induced β-catenin nuclear translocation. Thus, the transcriptional activity of TCF was partly suppressed, and concomitantly the expression of Wnt target genes was reduced by Lats2. In summary, our data show that Lats2 inhibits Wnt signaling to repress proliferation and accelerate differentiation of adipocytes.

In conclusion, Lats2 is an important modulator of adipose development, as it regulates the balance between proliferation and differentiation of adipocytes (Fig. S2A). Lats2 acts as a rheostat to control adipogenesis by inhibiting proliferation while accelerating differentiation of adipocytes via the Hippo and Wnt pathways (Fig. S2B).

## Supporting Information

Supplemental Figure S1 **Lats2 was successfully overexpressed in 3T3L1 preadipocytes and localizes mainly to the cytoplasm.** (A) Western blot analysis. Total cell lysates were prepared from Lats2-transfected 3T3L1 cells, immunoblotted with Lats2 and Tubulin antibodies, and compared with lysates from the Vector and Control (without any treatment) cells. (B) *Left*, a micrograph depicting Lats2 in 3T3L1 cells as detected by anti-Lats2 antibody (green). Note that Lats2 localizes to both the nucleus and cytoplasm, but mainly to the cytoplasm. *Middle*, micrograph showing nuclear staining by DAPI (blue). *Right*, merged micrographs demonstrating Lats2 and nuclei. The scale bar represents 20 µm.
(TIF)

Supplemental Figure S2 **Model for Lats2-mediated inhibition of adipocyte proliferation and promotion of adipocyte differentiation via Hippo signaling.** (A) Lats2 regulates the balance between cell proliferation and differentiation during adipose development. (B) Upon activation, Lats2 phosphorylates YAP and TAZ, leading to their retention in the cytoplasm and subsequent inability to bind to TEAD in the nucleus to activate its transcriptional activity. Thus, the expression of TEAD target genes (such as cyclin E, BIRC5 and CTGF) is repressed. Meanwhile, as TAZ remains in the cytoplasm and does not form a suppressive complex with PPARγ, PPARγ resumes its transcriptional activity to activate aP2/FABP4, C/EBPα and other genes. Interestingly, the cytoplasmic p-TAZ is not inactive but rather acts as an inhibitor of Wnt signaling by binding to DVL2 and suppressing DVL2 phosphorylation. Consequently, DVL2 does not protect β-catenin from destruction, and β-catenin does not enter the nucleus to co-activate TCF/LEF-mediated transcription, leading to the blockage of Wnt signaling. All of these mechanisms contribute to the phenotype of repressed proliferation and accelerated differentiation of adipocytes observed upon Lats2 overexpression.
(TIF)
