# Supplementary figures and images for: Lats2 Modulates Adipocyte Proliferation and Differentiation via Hippo Signaling
Source: PLoS One. 2013 Aug 16;8(8):e72042. doi: 10.1371/journal.pone.0072042 (PMC3745423; doi:10.1371/journal.pone.0072042)

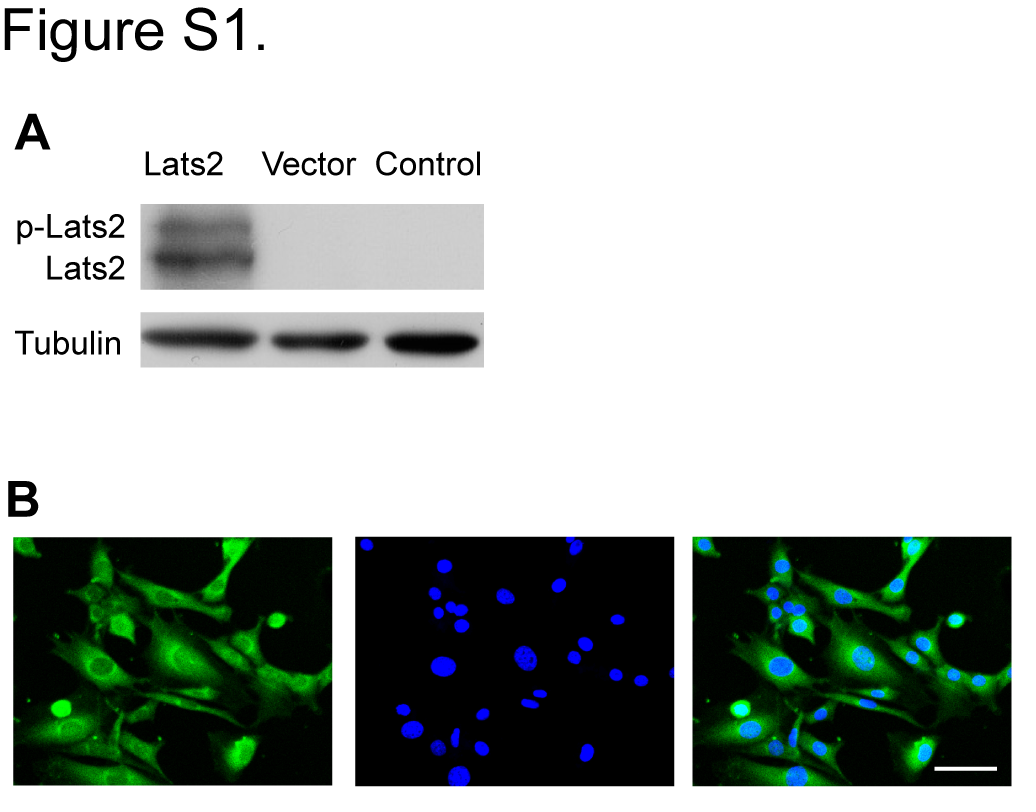

Supplement: Supplemental Figure S1 — Lats2 was successfully overexpressed in 3T3L1 preadipocytes and localizes mainly to the cytoplasm. (A) Western blot analysis. Total cell lysates were prepared from Lats2-transfected 3T3L1 cells, immunoblotted with Lats2 and Tubulin antibodies, and compared with lysates from the Vector and Control (without any treatment) cells. (B) Left, a micrograph depicting Lats2 in 3T3L1 cells as detected by anti-Lats2 antibody (green). Note that Lats2 localizes to both the nucleus and cytoplasm, but mainly to the cytoplasm. Middle, micrograph showing nuclear staining by DAPI (blue). Right, merged micrographs demonstrating Lats2 and nuclei. The scale bar represents 20 µm. (TIF) [file pone.0072042.s001.tif]

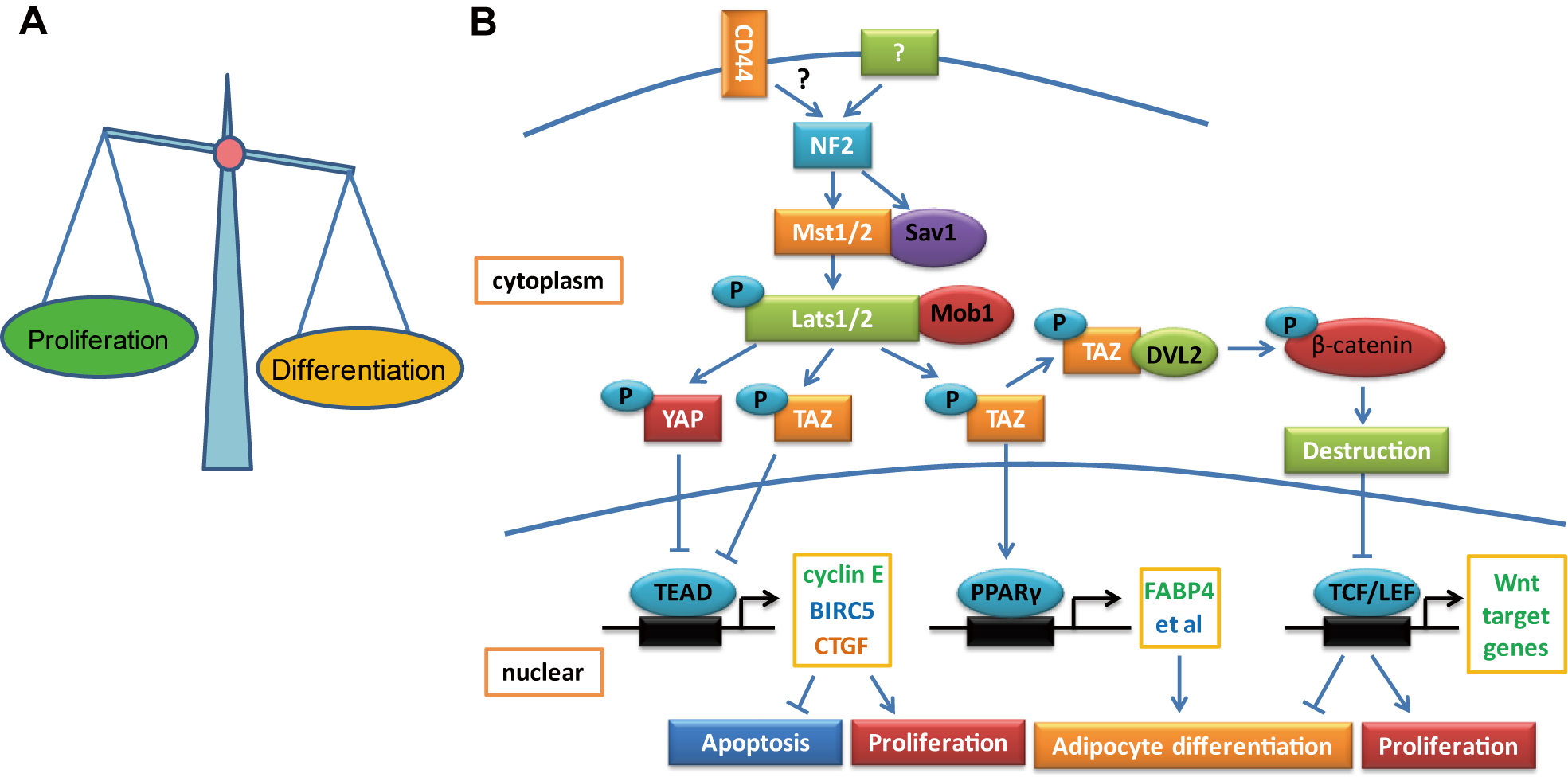

Supplement: Supplemental Figure S2 — Model for Lats2-mediated inhibition of adipocyte proliferation and promotion of adipocyte differentiation via Hippo signaling. (A) Lats2 regulates the balance between cell proliferation and differentiation during adipose development. (B) Upon activation, Lats2 phosphorylates YAP and TAZ, leading to their retention in the cytoplasm and subsequent inability to bind to TEAD in the nucleus to activate its transcriptional activity. Thus, the expression of TEAD target genes (such as cyclin E, BIRC5 and CTGF) is repressed. Meanwhile, as TAZ remains in the cytoplasm and does not form a suppressive complex with PPARγ, PPARγ resumes its transcriptional activity to activate aP2/FABP4, C/EBPα and other genes. Interestingly, the cytoplasmic p-TAZ is not inactive but rather acts as an inhibitor of Wnt signaling by binding to DVL2 and suppressing DVL2 phosphorylation. Consequently, DVL2 does not protect β-catenin from destruction, and β-catenin does not enter the nucleus to co-activate TCF/LEF-mediated transcription, leading to the blockage of Wnt signaling. All of these mechanisms contribute to the phenotype of repressed proliferation and accelerated differentiation of adipocytes observed upon Lats2 overexpression. (TIF) [file pone.0072042.s002.tif]
